# Reinforcement learning for closed-loop regulation of cardiovascular system with vagus nerve stimulation: a computational study

**DOI:** 10.1088/1741-2552/ad48bb

**Published:** 2024-06-03

**Authors:** Parisa Sarikhani, Hao-Lun Hsu, Mahmoud Zeydabadinezhad, Yuyu Yao, Mayuresh Kothare, Babak Mahmoudi

**Affiliations:** 1 Department of Biomedical Informatics, Emory University, Atlanta, GA, United States of America; 2 Department of Biomedical Engineering, Georgia Institute of Technology, Atlanta, GA, United States of America; 3 Department of Chemical & Biomolecular Engineering, Lehigh University, Bethlehem, PA, United States of America

**Keywords:** closed-loop VNS, reinforcement learning, neuromodulation, intelligent systems

## Abstract

*Objective*. Vagus nerve stimulation (VNS) is being investigated as a potential therapy for cardiovascular diseases including heart failure, cardiac arrhythmia, and hypertension. The lack of a systematic approach for controlling and tuning the VNS parameters poses a significant challenge. Closed-loop VNS strategies combined with artificial intelligence (AI) approaches offer a framework for systematically learning and adapting the optimal stimulation parameters. In this study, we presented an interactive AI framework using reinforcement learning (RL) for automated data-driven design of closed-loop VNS control systems in a computational study. *Approach.* Multiple simulation environments with a standard application programming interface were developed to facilitate the design and evaluation of the automated data-driven closed-loop VNS control systems. These environments simulate the hemodynamic response to multi-location VNS using biophysics-based computational models of healthy and hypertensive rat cardiovascular systems in resting and exercise states. We designed and implemented the RL-based closed-loop VNS control frameworks in the context of controlling the heart rate and the mean arterial pressure for a set point tracking task. Our experimental design included two approaches; a general policy using deep RL algorithms and a sample-efficient adaptive policy using probabilistic inference for learning and control. *Main results.* Our simulation results demonstrated the capabilities of the closed-loop RL-based approaches to learn optimal VNS control policies and to adapt to variations in the target set points and the underlying dynamics of the cardiovascular system. Our findings highlighted the trade-off between sample-efficiency and generalizability, providing insights for proper algorithm selection. Finally, we demonstrated that transfer learning improves the sample efficiency of deep RL algorithms allowing the development of more efficient and personalized closed-loop VNS systems. *Significance.* We demonstrated the capability of RL-based closed-loop VNS systems. Our approach provided a systematic adaptable framework for learning control strategies without requiring prior knowledge about the underlying dynamics.

## 1. Introduction

Cardiovascular diseases (CVDs) pose a significant health problem and financial burden [1]. The primary cause of death attributed to CVDs in the US is coronary heart disease, followed by stroke, high blood pressure, heart failure, diseases of the arteries, and other CVDs, as per the annual statistical report by the American Heart Association [2]. The insufficient effectiveness of the existing pharmaceutical therapies in treating CVDs has motivated research into alternative therapeutic options. Vagus nerve stimulation (VNS) has been identified as a potential treatment for various cardiac conditions, including heart failure, hypertension, atrial fibrillation, and stroke [3, 4].

VNS refers to delivering electrical stimulation to the vagus nerve through a pulse generator and is characterized by various stimulation parameters that include current amplitude (mA), pulse width (ms), and pulse frequency (Hz). One major challenge in delivering effective VNS therapy is determining optimal VNS parameters that can produce the desired physiological response. Currently, the optimal VNS parameters are determined through an open-loop trial-and-error approach as was used in three recent clinical papers that studied the effectiveness of VNS in the treatment of heart failure [5–7]. Authors in [3, 8] provided a review of available evidence regarding the effectiveness of VNS for preventing heart failure and emphasized the lack of systematic approaches for optimizing the VNS parameters. Hence, there is a need to develop systematic approaches aimed at optimizing VNS parameters to maximize the therapeutic effects.

Closed-loop VNS strategies offer the advantage of systematically tuning and adapting the stimulation parameters. Previous studies used classical control theory approaches to investigate the utility of developing closed-loop VNS. Authors in [9] utilized a proportional-integral (PI) controller [10] for real-time regulation of instantaneous heart rate (HR) through closed-loop VNS. There have been other investigations exploring the use of PI controller for heart-rate regulations [11–13]. In addition, the utilization of state-space transition models was examined by [14] for the development of closed-loop VNS systems. While these classical control approaches have demonstrated their effectiveness in recent studies, they have inherent drawbacks that make them impractical for real-world physiological applications. For instance, PI controllers have limited controllability making them less effective in transient responses. Tuning complexity, high sensitivity to model parameters, and sub-optimal performance in non-linear systems are among other disadvantages of PI controllers [15, 16]. While model predictive control (MPC) has been shown to address some of the challenges of classical control algorithms, it still requires extensive tuning of parameters such as the prediction horizon and control weights to achieve optimal performance [17]. Moreover, MPC requires having access to an accurate model of the physiological dynamics, where inaccuracies in the model predictions can affect the controller’s performance [17]. In practice, it can be challenging to obtain an accurate model of the complex physiological dynamics, and uncertainties can lead to sub-optimal control. There is a need to design novel closed-loop VNS techniques to address these limitations.

Developing and prototyping novel closed-loop VNS strategies requires utilizing in-silico simulation environments for evaluating the performance of these closed-loop systems before deploying in *in-vivo* experimental setups. Computational models of cardiac system that account for the effects of VNS play a crucial role in developing simulation environments for effectively designing closed-loop VNS control strategies. However, a lack of the necessary variables to account for the physiological effect of VNS in most of the existing computational models makes it challenging to adopt these models for applications of closed-loop VNS. Previous research predominantly concentrated on the optimization of VNS parameters for a sole physiological signal (i.e. HR) and for only one VNS stimulation location [11–13]. A recent study conducted an in-silico study to develop a rat cardiac model subjected to VNS in multiple VNS locations and implemented a MPC framework for regulating multiple physiological signals, i.e. HR and mean arterial pressure (MAP) [18].

While developing computational models of the cardiac system under VNS guides the design of novel closed-loop VNS strategies, the selection of correct underlying dynamics, parameter tuning, and difficulty of evaluation of such models caused by the variability of fiber recruitment in the vagus nerve makes it a challenging task. Furthermore, the computational cost of simulating full-scale in-silico physiological models for real-time closed-loop control systems adds to the challenges. To address the challenges of developing a computational cardiac model, a recent study [19] employed a data-driven modeling approach using long short-term memory (LSTM) neural networks. Authors in [19] demonstrated the utility of an LSTM model by generating synthetic data from the computational cardiac model introduced in [18] and developed an MPC controller to regulate HR and MAP. However, this approach still requires access to a substantial amount of experimental data with sufficiently sampling the stimulation parameter space to accurately model the effects of VNS parameters on HR and MAP, which can be impractical due to the limitations of experimental data collection.

Some data-driven optimization strategies like Bayesian optimization [20] eliminate the need of having access to underlying equations of the system to develop automated closed-loop neuromodulation systems. Authors in [21] adopted Bayesian optimization in the context of optimal experimental design with closed-loop real-time functional magnetic resonance imaging (fMRI). Bayesian optimization has been successfully utilized for seizure control [22], optimizing the DBS parameters using fMRI data [23] and for minimizing rigidity [24] for Parkinson’s disease (PD) patients. Authors in [25, 26] used Bayesian optimization and safe Bayesian optimization to develop an automated DBS programming framework with safety constraints for tremor suppression in PD and essential tremor patients. Bayesian adaptive dual control was introduced to reduce the beta power in a computational model of PD [27]. These data-driven optimization strategies have been successfully implemented in various applications of closed-loop neuromodulation systems. However, they share a common underlying assumption regarding the objective function’s stationarity, implying that its behavior remains constant within the region of interest. This assumption is essential for the acquisition function to effectively estimate regions of high uncertainty and is not applicable to dynamic systems like the cardiovascular system.

In order to overcome the aforementioned challenges while still achieving effective closed-loop VNS control, this paper presented a novel interactive artificial intelligence (AI) framework using reinforcement learning (RL) which provides an automated data-driven approach to design closed-loop VNS control policies with minimal assumptions and without the need for prior knowledge about the underlying physiological dynamics of the cardiovascular system. In addition, our approach enables continuous learning, where the system can learn from its experiences and continuously improve its performance which makes it suitable for developing long-term adaptive and patient-specific therapies.

By integrating the recent advancements in developing biophyics-based models of the rat cardiovascular system under multi-location VNS [28], we developed multiple simulation environments with a standard application programming interface (API) to design, prototype, and evaluate the performance of the proposed data-driven closed-loop neuromodulation framework. The standard API is called Gymnasium (previously known as OpenAI Gym) [29] and is developed in Python. The original implementation of the biophysical models was in MATLAB, which proved prohibitively computationally expensive. To reduce the computational cost, we developed a data-driven surrogate of biophysics based computational models of the rat cardiovascular system uisng temporal convolutional neural networks (TCN) [30] in Python. TCNs introduce several advantages over the canonical recurrent neural networks, such as longer memory retention and ability to exploit parallelism which makes it more computationally efficient. This approach aims to reduce the computational complexity and provide a unified programming environment in Python.

In this paper, we tested our hypothesis that the proposed closed-loop VNS programming framework effectively learns the neuromodulation control task. We developed multiple simulation environments of healthy and hypertensive rat cardiovascular system in rest and exercise states and designed a set point tracking task to regulate HR and MAP. We introduced two approaches of experimental design to perform the set point tracking of HR and MAP in cardiovascular system. First, we designed a general policy to regulate the cardiovascular system (HR and MAP) using deep RL algorithms, i.e. soft actor-critic (SAC) [31] and proximal policy optimization (PPO) [32]. Additionlly, since sample efficiency is critical in the design of closed-loop neuromodulation systems, we designed a sample-efficient adaptive policy using a model-based RL algorithm known as probabilistic inference for learning and control (PILCO) [33], which represents a sample-efficient approach to policy search. Furthermore, we examined the adaptability of the proposed frameworks to variations in both the target set-point and the underlying dynamics of the environment. Finally, transfer learning [34] is employed to improve sample efficiency for deep RL algorithms.

## 2. Methods

### 2.1. Simulation environments

**Figure 1.**
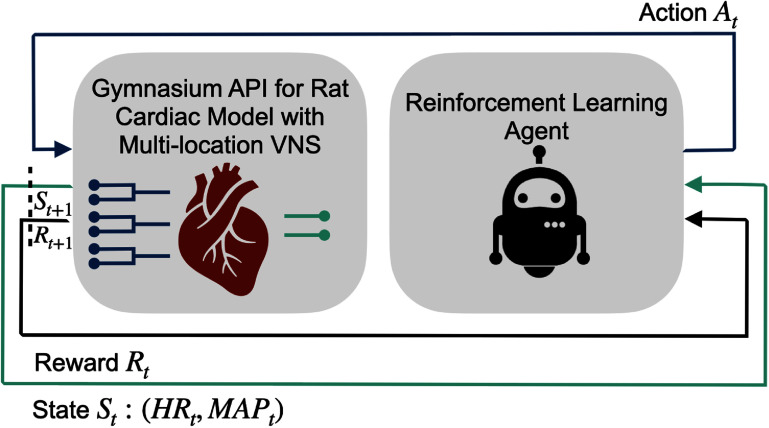
Overview of the architecture of the simulation environments for developing closed-loop VNS system demonstrating the interactions of the RL agent with the rat cardiac model using the standard API. The left block represents the reduced-order surrogates of the physiological cardiac models wrapped with the standard Gymnasium API, where the inputs of the model (color-coded as dark blue) are stimulation frequency and stimulation amplitude across three different locations at time $t$ (${A_t})$. The outputs of the model (color-coded as green) are the response of HR and MAP to the VNS parameters. The model estimates the response of the cardiac system (${\text{H}}{{\text{R}}_{t + 1}},{\text{ MA}}{{\text{P}}_{t + 1}}$) to the action ${A_t}$ taken at time step $t$ given the current state of the system (${\text{H}}{{\text{R}}_t},{\text{ MA}}{{\text{P}}_t})$. The right block represents the reinforcement learning agent, which takes action ${A_t}$ according to its policy at time step $t$, and observes the next state ${S_{t + !}}$, and reward ${R_{t + 1}}$.

**Figure 2.**
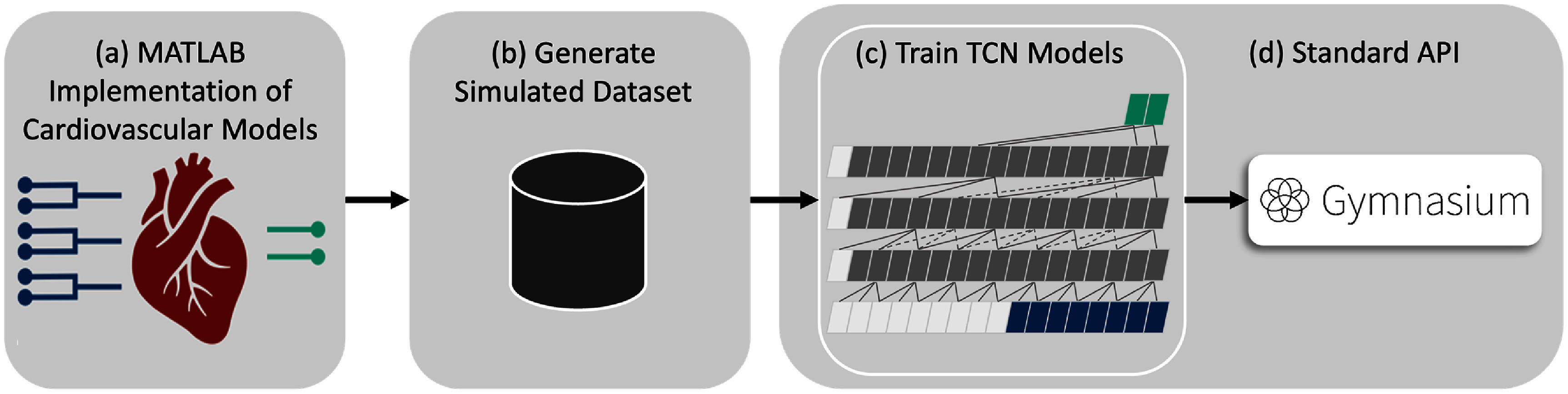
The pipline used for developing the simulation environments using the Gymnasium API to test and prototype RL algorithms for regulating the cardiovascular system. (a) Used the physiological models of rat cardiac system under multi-location VNS implemented in MATLAB, (b) generated a simulated data set of the response of the cardiac system by varying randomly selecetd VNS parameters, (c) trained the reduced-order TCN model to model the response of HR and MAP to VNS parameters, and (d) used the Gymnasium standard API wrapper over the trained TCN models for easier compatibility with RL algorithms.

We developed multiple simulation environments for evaluating the performance of the RL algorithms in developing closed-loop VNS systems. The high-level overview of the proposed closed-loop VNS system is depicted in figure 1. The pipeline of developing the simulation environments with the standard Gymnasium API is presented in figure 2. Detailed description of each component is provided in the following subsections.

#### 2.1.1. Standard API for rat cardiac model

We developed simulation environments of the rat cardiac model under multi-location VNS using a standard API called Gymnasium for testing and prototyping RL algorithm for regulating the cardiovascular system. Gymnasium (previously known as OpenAI Gym [29]) is a standard environment for developing and testing learning agents, especially RL agents. Gymnasium API is adopted for implementing the in-silico rat cardiac model to provide a standard interface for the users and provide the flexibility of designing their own control task with their learning agents of choice (figure 2(d)). The details of the in-silico physiological rat cardiac model are described in section 2.1.2.

#### 2.1.2. In silico rat cardiac model

We used a previously published physiological model of the integrated cardiovascular system and baroreflex regulation under multi-location vagal nerve stimulation [18]. The model was composed of three different parts including the cardiovascular system, the baroreflex system, and the VNS device. The cardiovascular model uses a lumped-parameter approach to predict the blood circulation in five elastic chambers representing the left heart, the arteries and veins in the upper and lower body. The right heart and the pulmonary circulation are modeled by capacitance, which is added to the venous capacitance in the upper body. The baroreflex system functions to regulate the arterial pressure through the baroreceptor, afferent pathway, efferent pathway and the effectors in cardiovascular system. Each of the compartments in the baroreflex system was modeled using a firing rate-based approach. The VNS device model predicts the response of firing rate of different fibers to VNS parameters [28, 35]. Three fiber types are engaged during VNS, representing the baroreceptive fibers, the vagal fibers, and the sympathetic fibers. Each type of fibers distributes nonhomogeneously in different locations. Activation of each fiber type in each stimulation location due to stimulation amplitude is modeled by an activation function, while the change of fiber activities due to stimulation frequency is represented by a conduction map. The overall physiological model of the rat cardiac system models the effect of VNS parameters (stimulation amplitude and frequency) in three different locations (leading to six total VNS parameters) on two physiological variables (HR (BPM) and MAP (mmHg)). The short-term effect of VNS parameters on the output of HR and MAP was calculated through the interactions between the cardiac systems and the neural regulation system.

To simulate the effect of VNS on MAP and HR in rats, the underlying parameters of the models (i.e. parameters representing the midpoint and slope of the activation curve, the weighing matrices of the conduction map, and the fiber distributions) were identified to match the previously collected experimental data [36, 37]. As shown in [28], the parameters of the activation curve varied with both fiber type and stimulation location since the distance between the cuff electrode and the target fiber may vary between different stimulation locations. The analysis of how MAP and HR react to changes in stimulation frequency and amplitude at a baroreceptive location revealed that stimulation parameters lowered the MAP, and only high amplitudes resulted in noticeable bradycardia [28]. The recruitment of barofibers, requiring a low amplitude, primarily contributed to the reduction in MAP, whereas the recruitment of vagal fibers was primarily responsible for the HR decrease observed with high amplitude. Furthermore [28], evaluated the effect of stimulation at a non-baroreceptive location on MAP and HR, finding that such stimulation slightly increased MAP but caused significant bradycardia. Hence the combination of multi-location VNS was required to induce the desired response. The increase in MAP can be attributed to the recruitment of sympathetic fibers, with 12% of the total sympathetic fibers and 50% of the total vagal fibers designated for the non-baroreceptive location. The third stimulation site is allocated the remaining 40% of the total vagal fibers. The development of the rat cardiac models with multi-location VNS are based on a strong assumption that three stimulation locations are isolated, meaning that the fibers in one location cannot be recruited by other locations. We refer the reader to [18, 28] for more details on the physiological model.

A modification of the described physiological model [28] is used in this study to simulate the physiological model in four different conditions adding the effect of hypertension and physical exercise (healthy and hypertensive rat cardiac models in rest and exercise states) by changing the related internal states and parameters. We refer to the four models as healthy cardiac environment (HC Env), healthy cardiac environment with the effect of exercise (HCE Env), hypertensive cardiac environment (HTC Env), and hypertensive cardiac environment with the effect of exercise (HTCE Env). Hypertension is related to increased arterial stiffness, vascular remodeling, increased sympathetic activities and decreased vagal activities. An offset in sympathetic and vagal activity coupled with modifications on the gain of each effector are used to represent the hypertensive condition. An acute exercise triggers multiple physiological responses, including redistribution of blood flow and modification of sympathetic and vagal activities by central command. An additional offset caused by exercise on sympathetic and vagal pathway, as well as the separation of the peripheral resistance into resting muscle resistance and active muscle resistance are used to represent the exercise condition. The new steady state during exercise consists of a higher arterial pressure, HR, stoke volume and cardiac output compared with rest state in both healthy and hypertensive condition.

#### 2.1.3. Reduced order model of the physiological rat cardiac model using TCNs

The physiological model of the rat cardiac system described in the previous section originally was implemented in MATLAB using the dde23 solver [38] which is computationally expensive. In addition, since the standard API for RL algorithms (Gymnasium) as well as most of the common RL algorithm libraries are developed in Python, we developed data-driven reduced-order models of the physiological models using TCN [30] to not only reduce the computational complexity, but also to provide a unified programming environment in Python.

The four physiological models were used to generate a dataset by varying the VNS parameters (stimulation amplitude and frequency) across the three VNS locations (leading to a six-dimensional parameter space) and measuring the effect of VNS parameters on HR and MAP (figures 2(a) and (b)). The range of VNS parameters in generating the synthetic data was $0{-}50{\text{ Hz}}$ for stimulation frequency and $0{-}1$ mA for stimulation amplitude. The response of HR and MAP to randomly selected VNS stimulation parameters was collected in 2000 runs. Each run consisted of 30 cardiac cycles to generate the dataset. The data was divided into training and test sets with an 80%–20% split. The training set was then used to train a reduced-order model using TCN (figure 2(c)). A TCN model with an input layer of width 8 and output layer of width 2, and three hidden layers of width 16 with the dilation factors of 1, 2, 4 was used. The standard API of Gymnasium (the maintained fork of OpenAI’s Gym library) was used to communicate between RL algorithms and the environments. The pipeline of developing the simulation prototyping of RL agents for regulating the cardiovascular function is depicted in figure 2.

### 2.2. Simulation experiments

#### 2.2.1. Regulating cardiovascular system using RL through designing a set point tracking task

Designing neuromodulation control systems depends on multiple factors including the computational and sample efficiency within the constraints of the problem, having access to related data sets or the equations of the underlying dynamics of the system, etc. Here, we assumed the underlying dynamics of the environment are unknown and hypothesized that utilizing RL eliminates this requirement while effectively learning to perform a set point tracking task for regulating HR and MAP values.

The subsequent subsections outline the simulation design employed to optimize the VNS parameters to regulate HR and MAP values during a set point tracking task. Specifically, two experimental design approaches were considered to elaborate the advantages and disadvantages of each method and provide guidance on future experimental design selection. Both experimental design approaches were used to perform the same set point tracking task, where the set point was a two-dimensional vector of desired HR and MAP values, and the agents were trained to apply proper stimulation parameters according to their policies with the goal of reaching the desired set points. The RL algorithm’s reward function was defined to perform the set point tracking task as described in section 2.3.4.

#### 2.2.2. Designing a general policy using deep RL algorithms

**Figure 3.**
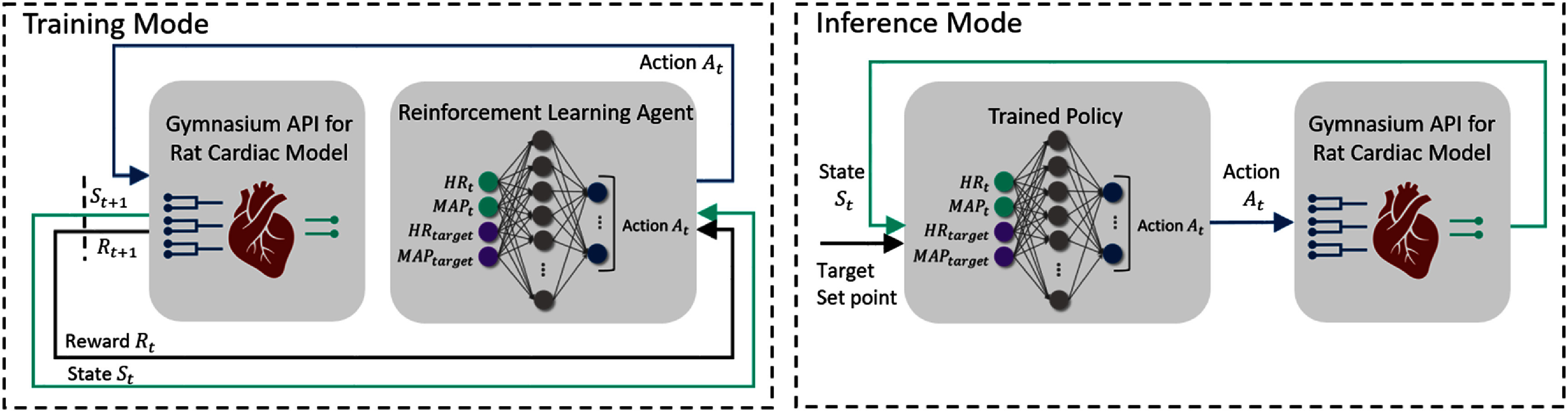
The overview of designing a general policy. The left panel represents the structure of the simulation environment with the standard Gymnasium API during the training mode. The right panel depicts the simulation environment in the inference mode. The policy network of the RL agents was designed as a simple MLP model, where ${\text{H}}{{\text{R}}_t}$ and ${\text{MA}}{{\text{P}}_t}$ are the current states of the environment. The input vector of the policy network was extended by adding ${\text{H}}{{\text{R}}_{{\text{target }}}}$ and ${\text{MA}}{{\text{P}}_{{\text{target }}}}$ (target set-points) to design a general policy. The environment is the reduced-order surrogate of the physiological cardiac models wrapped with the standard Gymnasium API, where the input of the model (color-coded as dark blue) are stimulation frequency and stimulation amplitude across three different locations at time $t$ (${A_t})$. The output of the model (color-coded as green) are the response of HR and MAP to the VNS parameters.

The first experimental design approach was to design a general policy to perform the set point tracking task, where the agent learned the general policy during the training mode and the trained policy was used in the inference mode to perform the set point tracking task. The overview of designing a general policy is depicted in figure 3. The term general is used to denote that the trained policy was designed to perform the set point tracking task for all of the potential set points within the possible range of HR and MAP values (table 1) rather than learning to follow a single set point at a time. Here, PPO and SAC algorithms as described in sections 2.3.1 and 2.3.2 were used to train a general policy. In both algorithms, the policy architecture was a fully connected feed-forward neural network also known as a multi-layer perceptron (MLP) [39]. We extended the input of the policy network to account for learning multiple set points at the same time (general policy) by feeding the desired set points ${\text{H}}{{\text{R}}_{{\text{target}}}}$ and ${\text{MA}}{{\text{P}}_{{\text{target}}}}$ as additional inputs to the policy network besides the current states of the environments (${\text{H}}{{\text{R}}_t}$ and ${\text{MA}}{{\text{P}}_t}$) as depicted in figure 3. During the training, each episode of length 500 was assigned to a randomly selected set point within the physiologically plausible range of HR and MAP values (table 1) for each of the four models. After training the RL agents, the policy network was used in the inference mode to perform the control task (right panel in figure 3).

**Table 1.** Sampling range of HR and MAP values ([minimum, maximum]) for the four cardiac environments.

	HC Env	HCE Env	HTC Env	HTCE Env
HR (BPM)	[234.4, 414.7]	[309.6, 578.6]	[245.8, 428.9]	[290.36, 578.1]
MAP (mmHg)	[71.9, 173.6]	[117.4, 158.1]	[86.6, 194.8]	[117.3, 178.3]

#### 2.2.3. Designing an adaptive policy using PILCO

**Figure 4.**
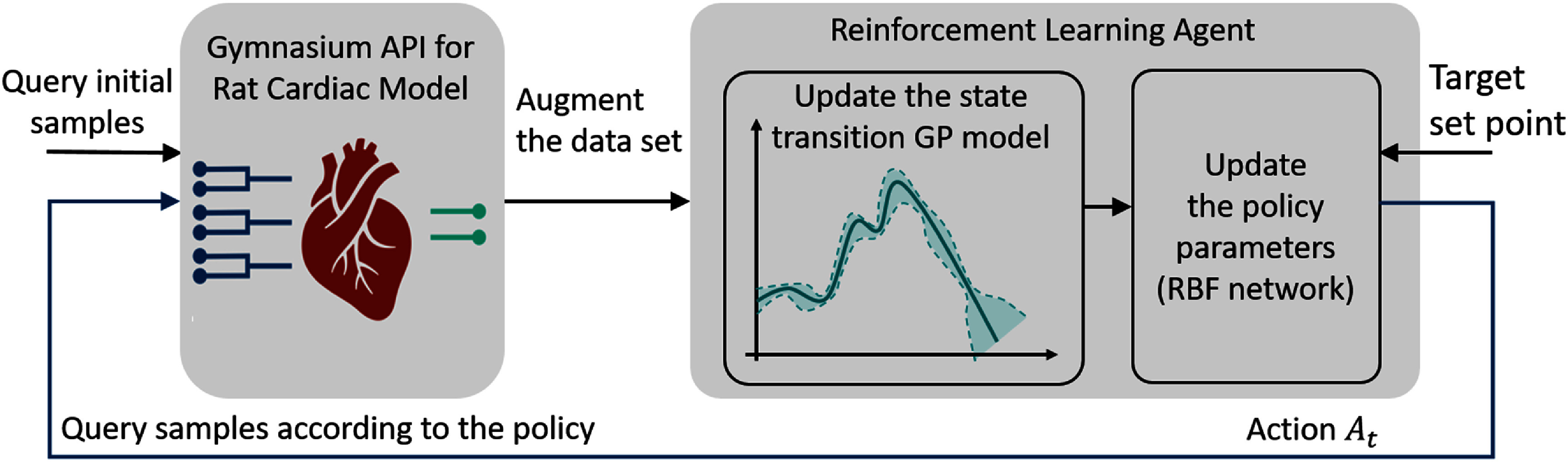
The workflow of adaptive policy using PILCO, illustrating the iterative process where actions are executed according to the recent policy (or randomly selected from the parameter space for the initial query) for N iterations. PILCO collects state transitions and reward values from the environment in response to the actions, augments its dataset, updates the Gaussian process (GP) model of the state transition, and adjusts the policy parameters based on the augmented data. This process is repeated to improve the adaptive policy over time. The environment is the reduced-order surrogate of the physiological cardiac models wrapped with the standard Gymnasium API, where the input of the model (color-coded as dark blue) are stimulation frequency and stimulation amplitude across three different locations at time $t$ (${A_t})$. The output of the model (color-coded as green) are the response of HR and MAP to the VNS parameters.

The second experimental approach aimed to dynamically learn an adaptive policy through interactive engagement with the environment (see figure 4). In this approach, we utilized PILCO, as described in section 2.3.3, to train the adaptive policy on-the-fly. PILCO operates by executing actions based on its policy for *N* iterations, gathering state transitions and reward values from the environment, augmenting its dataset, updating the underlying Gaussian process (GP) [40] model of the state transition, and adjusting its policy parameters using the augmented data and repeats the same process. The key distinction in designing an adaptive policy, as opposed to a general policy, lies in its ability to learn and track a specific setpoint during interactions with the environment. In contrast, a general policy in the inference mode can be used without further training to track multiple setpoints.

### 2.3. RL agents

A standard RL task can be formulated as a Markov decision process defined by a tuple $\left( {S,{\text{ }}A,{\text{ }}R,{\text{ }}T,{\text{ }}P} \right)$, where $S{\text{ }}$ and $A$ are state and action spaces, $R$ is a reward function (${R_t} = f({s_t},{\text{ }}{a_t},{\text{ }}{s_{t + 1}}$)), $T$ is the set of terminal conditions, and $P$ is the state transition probability. The goal of RL is to find the optimal policy ${\pi ^*}$ by maximizing the cumulative reward, typically with the discount factor $\gamma $. The overview of the standard RL framework is depicted in figure 1. The closed-loop flow of the interactions for the environment with the RL agents is as follows. At each time step, the agent interacts with the environment to learn the policy purely from interactions and without requiring prior knowledge about the underlying dynamics of the environment. The agent observes the current state at time $t$ and then takes an action with respect to the policy. Next, the environment returns the next state and reward at time step $t + 1$ (${S_{t + 1}},{\text{ }}{R_{t + 1}}$). This information is used to improve the policy. In this study, we employed two deep RL algorithms (PPO and SAC) to train a general policy and PILCO was employed to train an adaptive policy. The details of these algorithms are provided in the following sections.

#### 2.3.1. PPO algorithm

Policy gradient (PG) algorithms are a type of RL algorithms that rely upon optimizing parametrized policies with respect to the expected long-term return using gradient descent. Unlike vanilla PG [41] that keep new and old policies close in the parameter space, trust region policy optimization (TRPO) [42] algorithm updates policies by taking the largest step possible to enhance the performance while satisfying a constraint expressed in terms of KL-Divergence on how close the new and old policies are allowed to be. PPO combines the advantages of vanilla PG and TRPO to ensure stability and scalability by employing a surrogate objective function to update the policy parameters.

In this study, we employed PPO-Clip, a variant of PPO that utilizes specialized clipping in the objective function to prevent significant deviations of the new policy from the old policy. As a result, PPO offers a simpler implementation, while empirically performs at least as well as TRPO. PPO is applied in an actor-critic framework. The actor maps the state to action and the critic gives an expectation of the agent’s reward with its corresponding state. The policy is updated via a stochastic gradient ascent optimizer to ensure the exploration while the agent will gradually tend to exploit what it has learned over the course of training. A MLP model with input layer of width 4 (${\text{H}}{{\text{R}}_t},{\text{ MA}}{{\text{P}}_t},{\text{ H}}{{\text{R}}_{{\text{target}}}},{\text{ MA}}{{\text{P}}_{{\text{target}}}}$), one hidden layer of width 64, and output layer of width 6 (${A_t}$) was used to represent the policy network. Stable Baselines library [43] for implementing PPO algorithm was used in this study. The PPO algorithm was trained with a learning rate of 0.002, the rollout buffer size of 128, the discount factor of 0.95, the value function coefficient of 1, and the entropy coefficient for loss calculation of 0.005. A grid search-based approach was used to tune the parameters of PPO algorithm.

#### 2.3.2. SAC algorithm

SAC [31, 44] is a RL algorithm widely employed in continuous action spaces for various control tasks. SAC is from the family of off-policy RL algorithms that optimizes a stochastic policy. As the name suggests, SAC is also an actor-critic algorithm. A central feature of SAC is entropy regularization to encourage effective exploration during learning. The policy is trained to maximize a trade-off between expected return and entropy, a measure of randomness in the policy, which has a close connection to the exploration-exploitation trade-off. Increasing entropy results in more exploration, which can accelerate learning. It can also prevent the policy from prematurely converging to a bad local optimum, resulting in stable training. Moreover, SAC leverages neural networks to represent both the policy and the value functions, enabling it to handle high-dimensional observation spaces effectively. A MLP model with input layer of width 4 (${\text{H}}{{\text{R}}_t},{\text{ MA}}{{\text{P}}_t},{\text{ H}}{{\text{R}}_{{\text{target}}}},{\text{ MA}}{{\text{P}}_{{\text{target}}}}$), one hidden layer of width 64, and output layer of width 6 (${A_t}$) was used to represent the policy network. The same architecture of the policy network for both PPO and SAC algorithms was used. We employed Stable Baselines library [43] for implementing SAC algorithm. The SAC algorithm was trained with a learning rate of 0.000 03, a replay buffer size of 50 000, a mini batch size for each gradient update of 64, the soft update coefficient of 0.05, and a discount factor of 0.9. A grid search-based approach was used to tune the parameters of SAC algorithm.

#### 2.3.3. PILCO

PILCO is a model-based data-efficient approach to policy search [33], which offers improved sample-efficiency compared to model-free RL algorithms. Model-based RL algorithms rely on accurate models of the underlying dynamics of the system, which can result in reduced performance in the presence of model bias. Model bias is particularly an issue in cases where there is limited prior knowledge available. PILCO mitigates the need for prior access to the underlying dynamics of the environment by learning the model from observed data. Furthermore, PILCO utilizes GP, a non-parametric probabilistic model [40], to effectively address the issue of model bias by accounting for model uncertainty. The main advantage of PILCO is that it remarkably improves the sample efficiency in continuous state-action spaces which sets the pathway for the integration of PILCO in the implementation and deployment of closed-loop VNS systems in clinical settings and experimental setups.

Consider the following dynamics system

\begin{equation*}{x_t} = f\left( {{x_{t - 1}},{u_{t - 1}}{\text{ }}} \right),\end{equation*}

where $f$ is the unknown state transition function with continuous state, $x$, and action, $u$ domains. The goal of PILCO is to find a deterministic policy that maximizes the expected return or minimizes the expected cost, $c\left( {{x_t}} \right)$ of following the policy $\pi $ for $T$ time steps as in:

\begin{equation*}{J_\pi }\left( \theta \right) = \mathop \sum \limits_{t = 0}^T {E_{{x_t}}}\left[ {c\left( {{x_t}} \right)} \right],{x_0} \sim {\text{ }}N\left( {{\mu _0},{{{\Sigma }}_0}} \right).\end{equation*}

PILCO assumes that $\pi $ is a function parametrized by $\theta $ and that the cost function $c\left( x \right)$ encodes some information about a target state ${x_{{\text{target}}}}$. We used the squared exponential cost function as in the equation described in the next section. The GP model uncertainty is used for planning and policy evaluation steps. PILCO evaluates the policy using the deterministic approximate inference method which enables policy improvement through analytic PGs. Analytic PG is more efficient than estimating PGs with sampling and enables using standard non-convex optimization methods like LBFGS to find the optimal policy parameters. Here, the learned state-feedback controller is the nonlinear radial basis function network as follows:

\begin{align*}\pi \left( {x,\theta } \right) &amp;= \mathop \sum \limits_{i = 1}^n {\omega _i}{\phi _i}\left( x \right),\end{align*}

\begin{align*}{\phi _i}\left( x \right) &amp;= \exp {\bigg( { - \frac{1}{2} (x- {\mu _i}})^T}{{{\Lambda }}^{ - 1}}{\text{ }}\left( {x - {\mu _i}} \right)\bigg),\end{align*}

where the parameters of the RBF network controller were optimized using LBFGS optimization. We employed the implementation in this GitHub repository https://github.com/nrontsis/PILCO. In our simulations, the number of timesteps (*T*) for planning training and testing was set at 10, the number of rollouts before the beginning of optimization was set at 10. In addition, the optimization of the underlying GP model parameters was limited to a maximum of 100 iterations, while the controller optimization was capped at 20 iterations.

#### 2.3.4. Reward function

The exponential reward function was used in training all RL algorithms, where ${\text{ }}{x_t}$ was the two-dimensional state of the environment at time $t$ (${\text{H}}{{\text{R}}_t},{\text{ MA}}{{\text{P}}_t}$) and ${x_{{\text{target}}}}{ }$ was the two-dimensional target set-point of HR and MAP values (${\text{H}}{{\text{R}}_{{\text{target}}}}{\text{, MA}}{{\text{P}}_{{\text{target}}}}$). Here, the $\sigma _c^2{\text{ }}$ controls the width of $c$ and was set at 5 in our simulations

\begin{equation*}{ }c\left( {{x_t}} \right) = { }1 - {\text{exp}}\left( {{{\left| {\left| {{x_t} - {x_{{\text{target}}}}} \right|} \right|}^2}/\sigma _c^2} \right).\end{equation*}

#### 2.3.5. Transfer learning

Traditional machine learning approaches assume that the training and test data are in the same feature space and from the same distribution [34]. This requires having access to sufficient training data from the domain and distribution of interest which is not always practical. Transfer learning is a field that enables transferring the knowledge in a model developed for a particular task being reused as the starting point for a model on a second task, which not only improves the learning performance, but also reduces the need for expensive data collection and labeling [45]. This technique is particularly beneficial in scenarios where labeled data is scarce or expensive to obtain.

Developing data-driven closed-loop VNS systems is a challenging task and experimental data collection is expensive. Here, we leveraged transfer learning as a tool to improve the sample efficiency of deep RL algorithms. In addition to the improved sample efficiency, the model automatically learns to adapt to the changes in the underlying dynamics of the system. In this study, we initially trained a general policy on the HC rat model in resting state to learn the set point tracking task. We then used the trained policy in the HC model as an initialization of the policy for same set-point tracking task in the hypertensive rat model in resting state (HTC model). This approach aims to effectively transfer the knowledge learned from the healthy cardiac system to a hypertensive rat cardiac system, improving sample efficiency and facilitating the learning process in the hypertensive condition.

## 3. Results

### 3.1. Performance of TCN model

**Figure 5.**
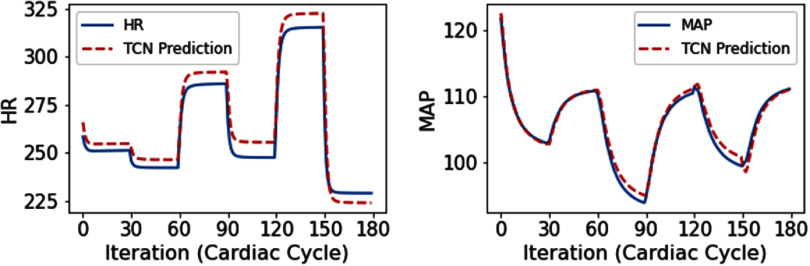
Comparison of the HR and MAP values predicted from the previously established biophysical model implemented in MATLAB versus the predictions from the reduced-order TCN model. The blue solid lines represent the HR and MAP values generated from the HC biophysical model implemented in MATLAB. The red dashed line represents the corresponding valued generated with reduced-order TCN model.

The four different rat cardiac models (i.e. the healthy and hypertensive models in rest and exercise states) were used to generate synthetic data for training a computationally more efficient TCN model as a surrogate of the previously established biophysical model [28] implemented in MATLAB. We evaluated the performance of the TCN models in predicting HR and MAP values as a function of stimulation parameters in three different locations. The normalized mean squared error (NMSE) of the predictions from the TCN models was calculated and reported in table 2. The NMSE is referring to the MSE calculated in the normalized state space. The normalized state space is calculated using the mean normalization approach for proper training of the RL algorithms. In addition, the computational efficiencies (how many times the predictions from the TCN model in the inference mode were faster than the same predictions from the biophysics model implemented in MATLAB) of the four reduced-order TCN models are reported in table 2. Our simulation of deep RL algorithms, using the reduced-order TCN model for 1000 episodes, required 278.72 ± 1.17 min for PPO and 411.70 ± 5.34 min for SAC in the HC environment. Conversely, using the previously established biophysical model implemented in MATLAB would have increased the computational time by a factor of 11.65, resulting in an average duration of 79.94 h for SAC. Additionally, a sample comparison of the HR and MAP values using the previously established biophysical model implemented in MATLAB versus the predictions from the reduced order TCN model is depicted in figure 5.

**Table 2.** Performance of the reduced-order TCN models; normalized mean squared error (NMSE) of TCN models on test set, and their computational efficiency compared to the previously established biophysical model implemented in MATLAB. The simulations were performed on a Linux Fedora system with Intel Core i7-8750 H @ 2.20 GHz × 12 CPU and 16 GiB memory.

	HC Env	HCE Env	HTC Env	HTCE Env
NMSE (HR)	0.002 07	0.002 55	0.003 80	0.002 51
NMSE (MAP)	0.000 08	0.000 64	0.000 77	0.001 43
Computational efficiency (*x* times faster than Matlab)	11.65	8.77	10.90	8.74

Furthermore, we conducted additional experiments in section 3.8 to evaluate the effect of measurement noise on the performance of the RL agent. The results in section 3.8 demonstrated that the RL agents’ performance remained stable by the noise levels at and below 5%, which confirms that the loss in accuracy using the reduced-order TCN model does not considerably affect the RL agents’ performance while improving the computational efficiency.

### 3.2. Performance of RL agents

**Figure 6.**
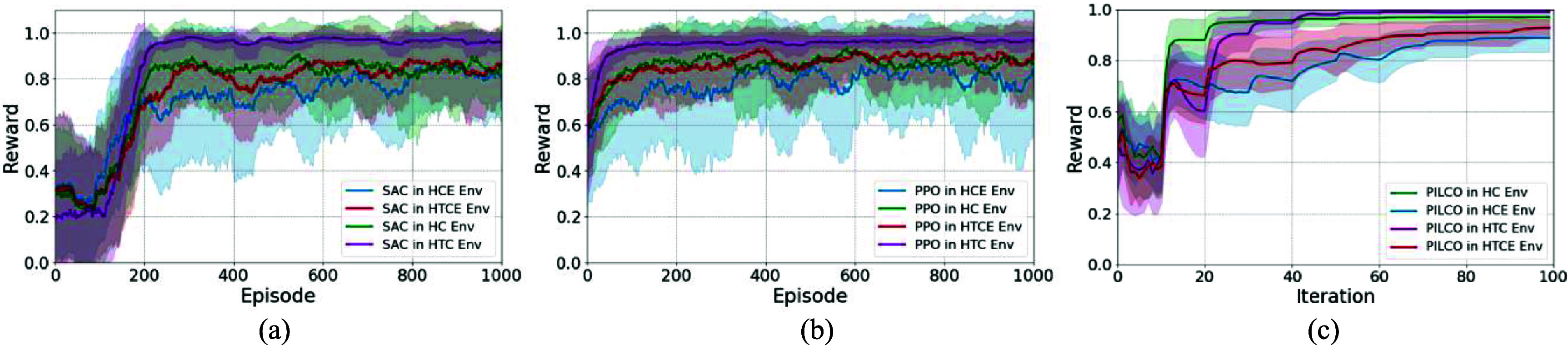
Reward values of the RL agents during the set-point tracking task in four cardiac environments; (a) normalized training reward values per episode for SAC during the training mode, (b) normalized training reward values per episode for PPO during the training mode, (c) reward values of PILCO for 20 randomly selected setpoints (mean ± standard deviation). The normalized reward for deep RL algorithms (a), (b) represent the mean ± standard deviation of the reward calculated through a moving average with window length of 50 to provide a better representation of the agents’ performances over time.

We conducted multiple experiments to evaluate and compare the performance of RL algorithms described in section 2.3 in performing the set-point tracking task for regulating HR and MAP values (figure 6). The normalized reward values of model-free deep RL algorithms (PPO and SAC) were demonstrated in figures 6(a) and (b). The total reward values per episode were normalized by the episode length of 500 and passed through an moving average function with window length of 50 to provide a better representation of the agents’ performances over time in four different environments (HC, HCE, HTC, and HTCE). As demonstrated in figure 6, PPO converges faster compared to SAC. The reward values of PILCO during the set-point tracking task for 100 iterations were depicted in figure 6(c) for the four rat cardiac models. As shown in figure 6, the convergence speed of PILCO is much faster than deep RL algorithms making it more suitable for experiments with a limited budget in terms of the number of interactions with the nervous system of interest.

**Figure 7.**
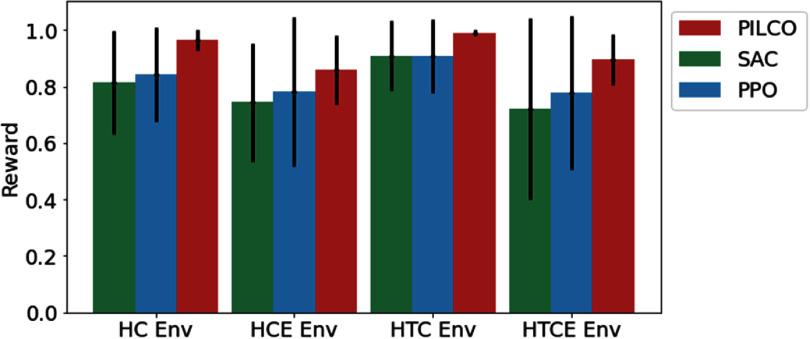
Performance comparison of the deep RL agents (PPO and SAC) during the inference mode and PILCO in four cardiac environments. The bars represent the average reward calculated over 20 randomly selected set points and the error bars (shown in back lines) represents the standard deviation of the set-point tracking performance across 20 randomly selected set points.

Additionally, we compared the performance of deep RL agents (PPO and SAC) in inference mode and PILCO in the four cardiac environments. The set-points are selected using a uniform distribution in the range of the minimum and maximum values of HR and MAP values for each environment (table 1). The barplots in figure 7 represent the mean ± standard deviation of the reward value during the set-point tracking task. The set-point tracking task is performed for each set-point for 100 cardiac cycles and the average and standard deviation of the rewards are calculated during the last 50 iterations to ignore the transient response and have a better estimate of the agents’ performance. The set-points are considered to be similar for all three algorithms for a fair comparison.

### 3.3. Performance of deep RL agents (PPO and SAC) in set point tracking task in four cardiac models

**Figure 8.**
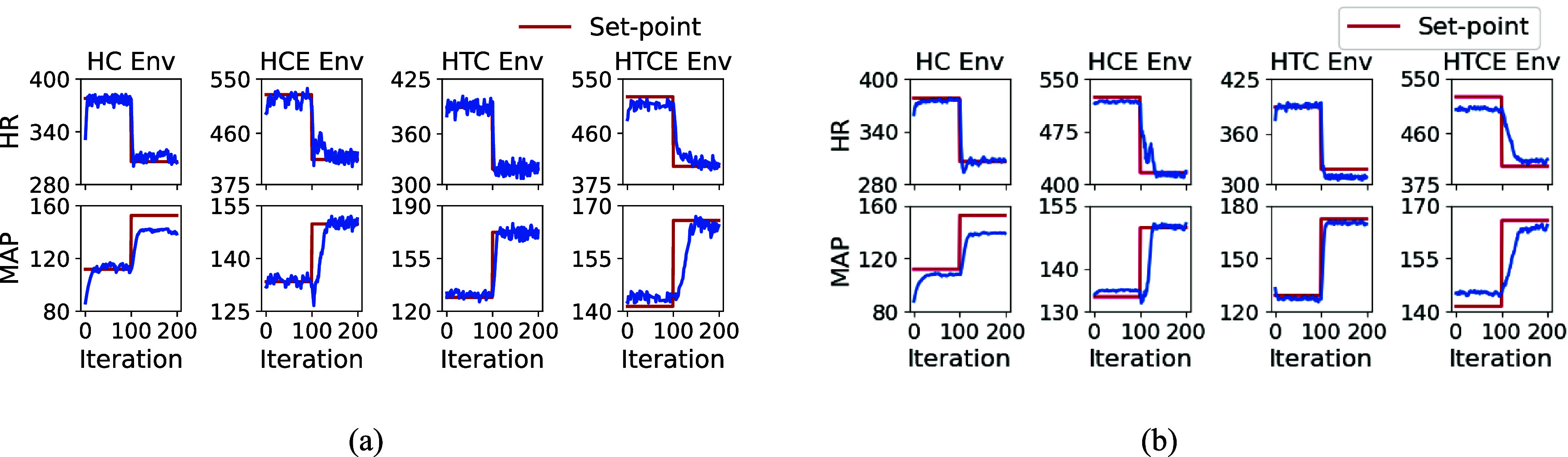
The performance of deep RL algorithms in inference mode for set-point tracking task across four cardiac environments using (a) PPO and (b) SAC algorithms for 200 iterations. The red solid lines represent the desired set points and the blue lines represent the states of the four cardiac models (HR and MAP). The target set points were changed after 100 iterations, where iterations are equal to the cardiac cycle.

**Figure 9.**
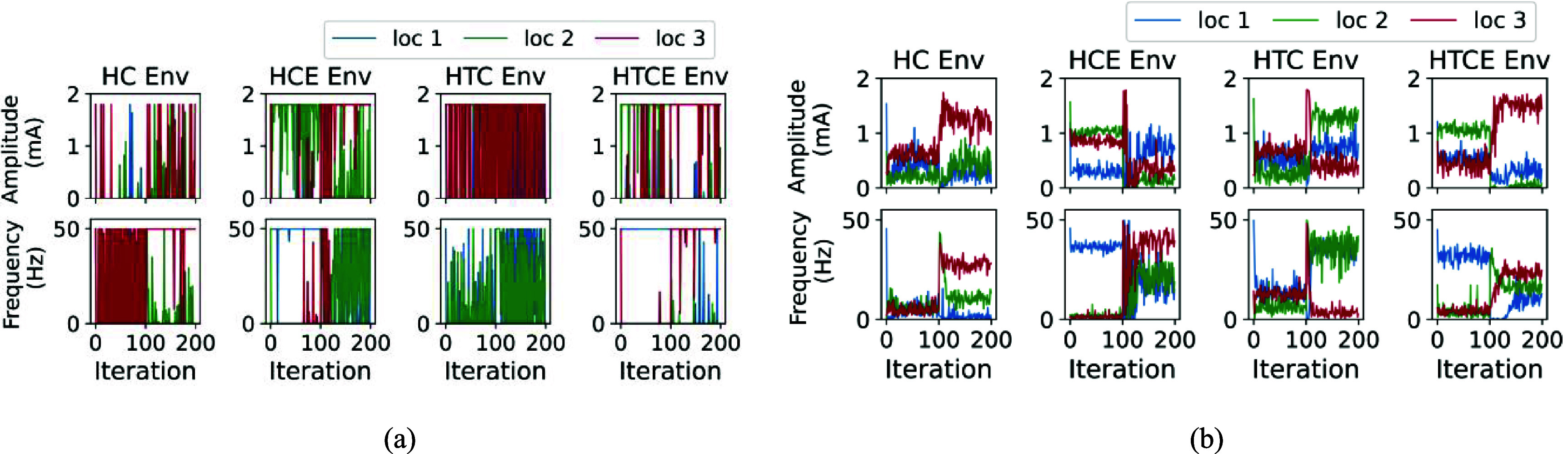
The stimulation parameters used during the inference mode for the set-point tracking task across the four cardiac environments (a) using PPO and (b) SAC algorithms for 200 iterations. The stimulation parameters were amplitude and frequency across three VNS locations. The target set points were changed after 100 iterations, where iterations are equal to the cardiac cycle.

The performance of deep RL algorithms in inference mode for set-point tracking task across four cardiac environment using PPO and SAC algorithms is depicted in figures 8(a) and (b), respectively. Their associated actions (stimulation parameters) taken during the inference mode were demonstrated in figures 9(a) and (b). The set-points in figure 8 are set to be 40% and 80% of the maximum range of HR and MAP for each of the four environments. As shown in the figures, the trained general policy in PPO and SAC algorithms learned to track the target set points.

### 3.4. Performance of PILCO in set-point tracking task in four cardiac models

**Figure 10.**
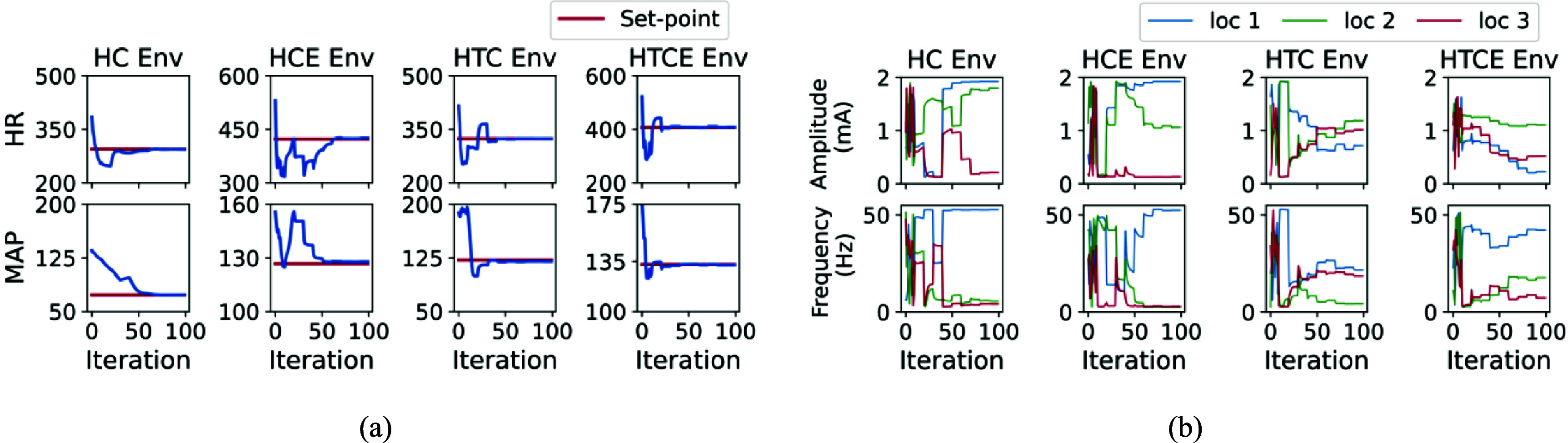
The performance of PILCO in set-point tracking task across four cardiac environments using PILCO (a) and its corresponding stimulation parameters (amplitude and frequency) across three stimulation locations (b). In the left figure (a), the red lines represent the desired set points and the blue lines represent the states of the four cardiac models (HR and MAP).

The performance of set-point tracking for the PILCO algorithm is depicted in figure 10(a). The associated actions (i.e. stimulation parameters) taken across the three VNS stimulation locations are demonstrated in figure 10(b). As shown in these figures, PILCO started by taking $N = 10$ random samples and gradually learned a GP model of the underlying dynamics as well as a RBF network policy to track the target set-point. As shown in figures 6(c) and 10(a) PILCO learns to track the target set point in less than 100 iterations.

### 3.5. Adaptability of PILCO to variations in target set-point

**Figure 11.**
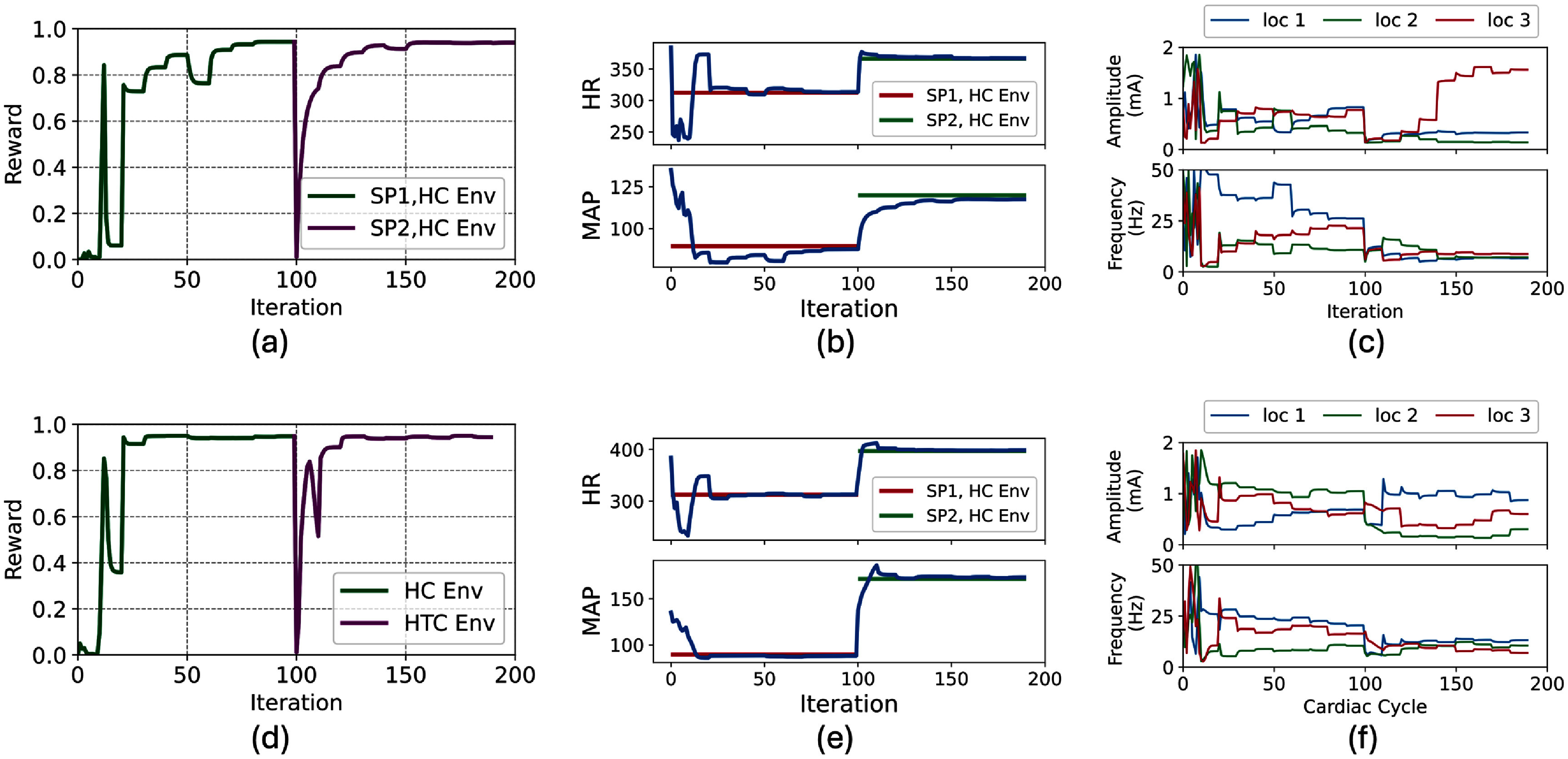
Adaptability of PILCO during the set point tracking task to variations in the target set point (a)–(c) and to variations in the underlying dynamics of the environment (d)–(f). (a), (d) reward value; (b), (e) the state trajectory, and (c), (f) stimulation parameters for 200 iterations, where the changes were applied after 100 iterations.

Unlike model-free deep RL algorithms (i.e. PPO and SAC) that were designed to train a general policy that has the ability to track a wide range of set points, PILCO is designed to track a single set point at a time. We designed an experiment to change the target set-point after the first 100 iterations to test the performance of PILCO in learning a new randomly selected set-point in the HC environment. The reward values for PILCO in adapting to a new target set point for the HC environment was provided in figure 11(a). As shown in figures 11(a)–(c), PILCO learned to track the new set point in around 40 iterations.

### 3.6. Adaptability of PILCO to variations in the underlying dynamics of the environment

Another experiment was designed to validate the ability of PILCO in performing the set-point tracking task when the underlying dynamics of the system changes over time. Non-stationarity of the underlying dynamics of the nervous system is a challenge that should be considered in the design of closed-loop neuromodulation systems. To simulate this scenario, after 100 iterations, we changed the environment from HC to HTC environment. The reward values for PILCO in adapting to a target set point in a new cardiac environment (HTC) after 100 iterations was provided in figure 11(d). As shown in figures 11(d) and (e), PILCO learned to track the new set point in the new environment in around 20 iterations.

### 3.7. Adaptability of PPO and SAC to variations in the underlying dynamics of the environment using transfer learning

An experiment was conducted to assess if PPO and SAC can adapt to changes in the underlying dynamics of the environment. The goal of this experiment was to simulate one of the challenges in *in-vivo* setups, where the physiological dynamics are not stationery and change over time. To achieve this, transfer learning was employed, and the pre-trained policy in the HC model was used as the initial policy instead of starting with a random policy. Transfer learning was adopted to fine-tune the model to perform the set-point tracking task in the HTC environment.

**Figure 12.**
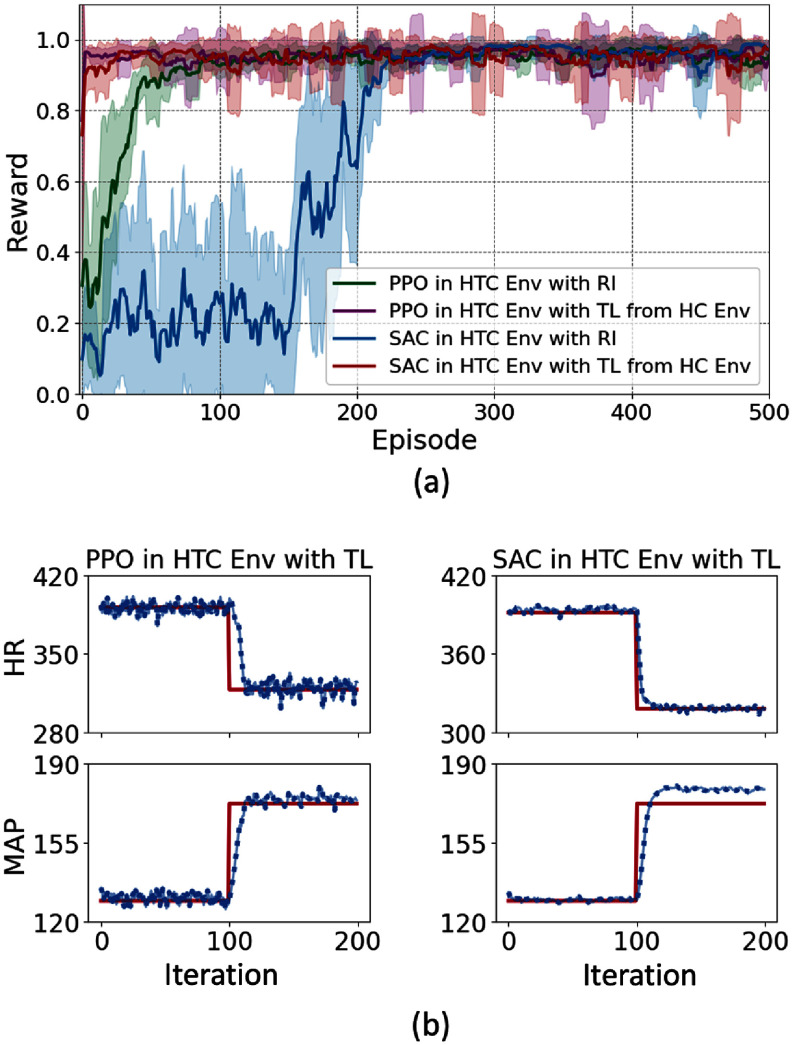
Adaptability of PPO and SAC algorithms to the variations in the underlying dynamics of the environment using transfer learning; (a) comparison of the reward values of PPO and SAC with random initialization (RI) and with transfer learning, (b) performance of PPO and SAC in set point tracking task with the trained policy using transfer learning approach.

As depicted in figure 12(a), transfer learning considerably improved the speed of convergence for both RL agents (PPO and SAC) and enabled them to quickly adapt to the new dynamics of the environment. SAC and PPO converged in less than 10 episodes with transfer learning as opposed to more than 200 and 100 episodes without transfer learning, respectively. The result of performing the set-point tracking task with the fine-tuned policy using transfer learning from the HC environment to HTC environment is depicted in figure 12(b).

### 3.8. Evaluating the effect of measurement noise on the RL agents’ performance

**Figure 13.**
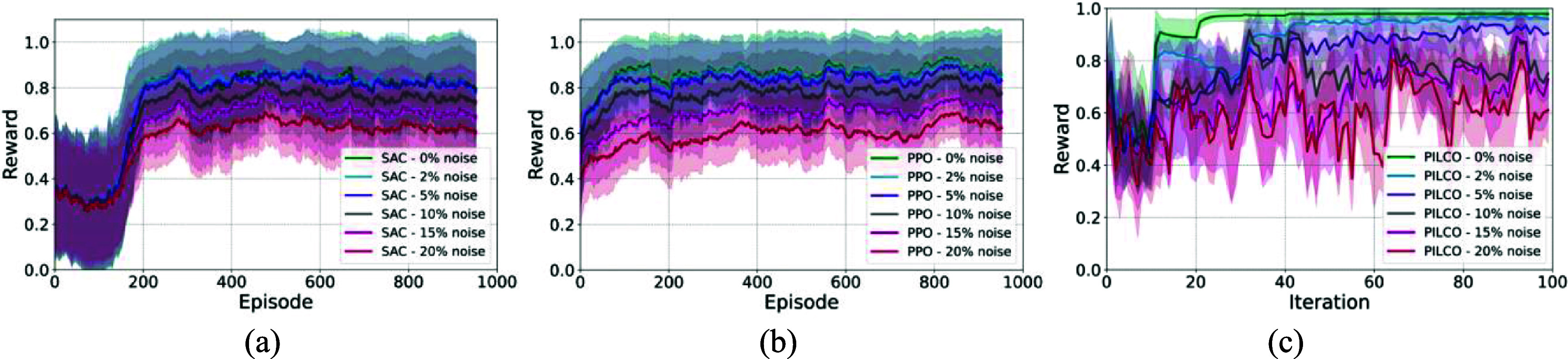
The effect of the presence of different levels of measurement noise on the performance of the RL agents. Each figure represents the reward values of RL agents during the set-point tracking task in the HC model with different levels of noise added to the state space; (a) normalized training reward values per episode for SAC during the training mode, (b) normalized training reward values per episode for PPO during the training mode, (c) reward values of PILCO during the experiment. The normalized reward for deep RL algorithms (a), (b) represent the mean ± standard deviation of the reward calculated through a moving average with window length of 50 to provide a better representation of the agents’ performances over time. The reward value of PILCO shows the mean ± standard deviation of the reward during 5 runs.

To evaluate the performance of RL agents in performing the set-point tracking task in the presence of measurement noise, we conducted an experiment by introducing various levels of noise to the state space (i.e. HR and MAP) of one representative rat cardiac model (HC model) during the training mode. Additive gaussian noise with zero mean and varying standard deviations (expressed as the percentage of the maximum range of the HR and MAP values) was added. As shown in figure 13, the RL agents’ performance remained stable and close to the noise-free scenario at low noise levels (2% and 5% of the maximum values of HR and MAP) and started to decline as the signal-to-noise ratio decreased. In these simulation experiments, the random seeds were fixed to isolate the effects of different noise levels from the other random factors.

## 4. Discussion

In this study, we described and evaluated an interactive AI framework using RL for automated data-driven design of closed-loop VNS control systems. We implemented this framework to regulate HR and MAP in computational models of rat cardiovascular system under multi-locations VNS. We provided multiple simulation environments using biophysics-based computational models of rat cardiovascular system in four different conditions including healthy and hypertensive rat in rest and exercise states (HC, HTC, HCE, and HTCE models). The simulation environments were built through the standard Gymnasium API (previously known as OpenAI gym) to facilitate testing and prototyping of the interactive RL-based closed-loop neuromodulation systems. Furthermore, we showed the utility of the framework in designing adaptive closed-loop neuromodulation systems through a set-point tracking task. In addition, two experimental design approaches (i.e. general policy and adaptive policy) feasible for the integration of RL algorithms were introduced which could be utilized based on the limitations and requirements of the application of interest. We compared the performance of the framework using multiple model-free and model-based RL algorithms in terms of sample efficiency and quality of performing the set-point tracking task as well as their ability to adapt to the new target set points and to the variations in the underlying dynamics of the environment.

We provided multiple simulation environments for testing and prototyping RL agents and designed a set-point tracking task to modulate the desired HR and MAP values. TCN was used to build reduced order models of the previously established biophysical models (which was implemented in MATLAB) in Python with the standard OpenAI Gym environment and our results confirmed the improved computational time (table 2) while providing a coherent programming environment in Python. Integrating the computational models of the cardiac system with VNS facilitates the testing and prototyping of novel closed-loop VNS strategies in simulation. However, a translational gap remains in the practical implementation of such systems. To narrow the translational gap, future model development efforts should aim at modeling realistic scenarios. This includes incorporating realistic stimulation signal waveforms, accurately reflecting the capabilities and limitations of the experimental devices, in addition to minimizing biologically unrealistic assumptions.

A control policy is at the core of an automated closed-loop neuromodulation system which automatically adjusts the stimulation parameters to achieve the goals of a desired neuromodulation task. Current approaches to adjusting VNS parameters are based on open-loop trial-and-error method [5, 6, 8] and a systematic approach of tuning VNS parameters is needed [8]. Recent studies investigated the utility of MPC in regulating HR and MAP values through VNS [18, 19], however, they required having access to an accurate model of the underlying dynamics which is not practical for many applications. Our results support the hypothesis that our interactive AI framework can generate effective VNS control policies in a data-driven fashion with minimal assumptions and without the requirement of having access to the exact underlying dynamics of the nervous system. This is particularly important for *in-vivo* experiments, where we do not have access to an underlying dynamical model of the system. Our framework enables continuous learning from experience which makes it suitable for developing long-term patient-specific therapies. Scalability to continuous state and action spaces, actively exploring to improve the performance and the ability to learn in real-time from data are among the other advantages of the RL-based control strategies. These capabilities make the proposed framework a suitable approach for designing adaptive *in-vivo* experiments.

Our automated and adaptive VNS programming framework was evaluated using multiple model-free and model-based RL algorithms. The comparison of PPO, SAC, and PILCO algorithms provides insights into the performance of different classes of RL approaches and guides the algorithm selection for the design of closed-loop VNS neuromodulation system. Model-free deep RL algorithms have shown to be less sample efficient than PILCO at the expense of learning a general policy. As shown in figure 6, PPO and SAC converge around 100 and 200 episodes (50 000 and 100 000 cardiac cycles), respectively to learn a general policy that learns to track any potential set point in the HR and MAP range (table 1). On the other hand, PILCO learns to track a single set point after around 50 cardiac cycles, making it more practical for *in-vivo* experimental setups. PPO, known for its stability and sample efficiency, demonstrated promising results in maintaining control over the set-point tracking task across different cardiac environments and over the range of potential target set points. SAC, with its emphasis on exploring the action space, exhibited competitive performance with a smoother behavior in the action space (figure 9). On the other hand, PILCO, an uncertainty-driven and model-based algorithm, showed robustness in handling the inherent variability in the neuromodulation system (underlying dynamics of the environment or the target set point) while requiring a considerably smaller number of samples to learn the set point tracking task.

In addition, two experimental design approaches were considered. First, we integrated deep RL algorithms to train a general policy capable of tracking a range of set points in the inference mode. Additionally, PILCO was used to train an adaptive policy on the fly, which is capable of tracking one predefined set of set points while being able to adapt to the variations in the target set point over time. As demonstrated in figure 7, PILCO outperforms the deep RL algorithms in inference mode in performing the set point tracking task over randomly selected set points in noise-free environment. However, PILCO demonstrated higher sensitivity to the higher levels of noise (figure 13). These findings highlight the trade-offs between sample efficiency, generalizability, adaptability, and robustness to noise in the context of closed-loop VNS neuromodulation, offering researchers and practitioners valuable insights for selecting the most suitable algorithm for their specific application requirements. Further research could explore hybrid approaches or algorithm modifications to enhance the performance of these algorithms in closed-loop neuromodulation systems.

While PILCO demonstrated promising performance in quickly learning the set point tracking task as well as in adaptability to the target set point and the variations in the underlying dynamics of the environment, one of the limitations of PILCO is the use of GP approach and its limited generalizability to higher dimensions. While GP modeling and PILCO have proven to be very sample-efficient effective in low-dimensional control problems, their performance tends to degrade as the dimensionality increases [33]. Therefore, future directions should explore alternative approaches that can enhance sample efficiency and improve generalizability to higher dimensions.

While deep RL algorithms are less sample efficient than PILCO, their ability to learn a general policy is valuable in the context of developing a generalized closed-loop VNS therapy with the capability of adapting to the unique needs of individual patients. Therefore, it is crucial to come up with potential approaches to improve their sample efficiency which facilitates their integration in clinical and experimental setups. To address this problem, we integrated transfer learning as a potential approach to improve sample efficiency of deep RL algorithms. By incorporating transfer learning, we can leverage pre-existing knowledge from related tasks or domains to initialize and guide the learning process of the deep RL algorithms. In this study, we trained a general policy using the healthy rat cardiac model in rest state (HC Env) and incorporated this prior knowledge by using transfer learning to improve sample efficiency for the hypertensive cardiac model in rest state (HTC Env). Our results demonstrated a considerable improvement in sample efficiency of deep RL algorithms using transfer learning (figure 12(a)). Transfer learning offers a promising avenue paving the way for the development of more efficient and personalized closed-loop VNS systems. Future research can further explore novel transfer learning techniques tailored specifically for closed-loop VNS systems to enhance their performance and usability in real-world scenarios.

A major challenge in translating computational studies into practical *in-vivo* experimental setups is the lack of consideration of real-world experimental issues including the noise and non-stationarity of the model. Here, we studied the effect of introducing varying levels on measurement noise (figure 13) and observed that the RL agents’ performance remained stable with lower levels of noise (2% and 5% of the maximum HR and MAP values) and dropped with increasing the noise level. Moreover, as illustrated in figure 13, PILCO, a model-based RL approach shows more sensitivity to the higher levels of noise. This increased sensitivity may stem from inaccuracies in the underlying GP models, that is used for stimulation planning, introduced by higher levels of noise. Another real-world experimental issue is the non-stationarity of the underlying dynamics of the nervous system. In this study, we did not consider non-stationarity of the models explicitly but evaluated the adaptability of the agents to the variations of the underlying dynamics over time in a broader sense (figures 11 and 12). Considering the non-stationarity in the biophysical model development, which seeks to mimic real experimental data, is a promising future direction. This approach enables developing controllers capable of adjusting to variable model dynamics in a more rigorous way. In addition, the simulation experiments could be expanded to account for various real-world experimental challenges. The expansion would consider the constraints of experimental setups, including the limitations of real stimulation instruments and safety constraints. Safety considerations is an important aspect in practical deployment of RL-based closed-loop neuromodulation systems. Integrating prior knowledge to define safe parameter space, using safety-constrained RL and optimization approaches, and incorporating interpretable RL techniques are among the promising future directions. Defining alternative reward functions to account for desired safety consideration, e.g. smooth variations in the stimulation parameters is an alternative approach. Integrating these approaches will further reduce the translational gap from in-silico studies to *in-vivo* experiments.

## 5. Conclusion

In this study, we developed and evaluated an interactive AI framework using RL for automated data-driven design of closed-loop VNS control systems. Multiple simulation environments were created to model different cardiac conditions, and RL algorithms, including PPO, SAC, and PILCO, were employed to autonomously learn the set-point tracking tasks. Our results confirmed that the proposed interactive closed-loop VNS control framework offer a data-driven alternative to classical control methodologies, allowing for continuous learning and the development of precision neuromodulation therapies that autonomously learn and adapt to the underlying dynamics of the cardiovascular system. The integration of transfer learning was found to improve the sample efficiency of deep RL algorithms, offering the potential for the development of more efficient and personalized closed-loop VNS systems.

## Data Availability

All data that support the findings of this study are included within the article (and any supplementary files).
